# The Effect of Auricular Acupoint Stimulation in Overweight and Obese Adults: A Systematic Review and Meta-Analysis of Randomized Controlled Trials

**DOI:** 10.1155/2017/3080547

**Published:** 2017-12-05

**Authors:** Tzu-Lin Yeh, Hsin-Hao Chen, Tsung-Ping Pai, Shu-Jung Liu, Shang-Liang Wu, Fang-Ju Sun, Lee-Ching Hwang

**Affiliations:** ^1^Department of Family Medicine, Hsinchu MacKay Memorial Hospital, No. 690, Section 2, Guangfu Road, East District, Hsinchu City, Taiwan; ^2^Department of Family Medicine, Taipei MacKay Memorial Hospital, No. 92, Section 2, Zhongshan North Road, Taipei City, Taiwan; ^3^Department of Medical Library, MacKay Memorial Hospital, Tamsui Branch, No. 45, Minsheng Road, Tamsui District, New Taipei City, Taiwan; ^4^School of Medicine, Griffith University, Gold Coast Campus, Parklands Dr., Southport, QLD 4215, Australia; ^5^Department of Medical Research, MacKay Memorial Hospital, No. 92, Section 2, Zhongshan North Road, Taipei City, Taiwan

## Abstract

**Objective:**

To investigate the effect of auricular acupoint stimulation on overweight and obese adults.

**Methods:**

We searched databases including PubMed, EMBASE, Allied and Complementary Medicine Database, National Knowledge Infrastructure, and the PerioPath Index to Taiwan Periodical Literature. The modified Jadad scale was used to assess study quality. We investigated the effect of auricular acupoint stimulation on anthropometric measurements.

**Results:**

Eighteen randomized controlled trials (RCTs) were included in our systematic review. Thirteen RCTs were pooled in a meta-analysis that revealed a significant reduction in body weight (BW) with a mean difference (MD) of −1.21 kg and a 95% confidence interval (CI) from −1.94 to −0.47 with a heterogeneity of *I*^2^ = 88%. Significant decreases in body mass index (BMI; MD: −0.57 kg/m^2^; 95% CI −0.82 to −0.33; *I*^2^ = 78%), body fat (BF; MD: −0.83%; 95% CI −1.43 to −0.24; *I*^2^ = 0%), and waist circumference (WC; MD: −1.75 cm; 95% CI −2.95 to −0.55; *I*^2^ = 87%) were also revealed.

**Conclusions:**

This meta-analysis shows that auricular acupoint stimulation improves physical anthropometric parameters including BW, BMI, BF, and WC in overweight and obese adults. These methods are less effective on hip circumference and waist-to-hip ratio.

## 1. Introduction

Obesity is an increasingly common chronic disease worldwide. Overweight and obesity represent a rapidly growing threat to the health in an increasing number of countries [1]. The prevalence of adult obesity in the United States was 34.9% based on data collected by the National Health and Nutrition Examination Survey between 2011 and 2012 [2]. Obesity and increased central fat increase the relative risk of hypertension, hypercholesterolemia, and diabetes mellitus [3] and increase morbidity and mortality [4, 5]. The basic anthropometric measurements for evaluating overweight and obese patients are body height, body weight (BW), body mass index (BMI), body fat (BF), waist circumference (WC), hip circumference (HC), and waist-to-hip ratio (WHR). Anthropometric measurements are reliable physical parameters to evaluate the effects of weight loss.

Weight loss provides a number of cardiac [6, 7] and noncardiac benefits [8], and a number of complementary therapies are used to treat overweight and obese individuals. Auricular acupoint stimulation, also called ear stimulation or auriculotherapy, is a method of diagnosing and treating physical and psychosomatic dysfunctions by stimulating a specific point in the ear. Many methods are used, such as finger acupressure, electrical stimulation, lasers, different types of needles, seeds, and magnetic balls [9]. Of these, auricular acupuncture by needle is the most common. Auricular acupuncture is a convenient method used to treat many conditions, such as substance abuse, pain, obesity, anxiety, epilepsy, and sleep disorders. Both experimental and clinical data suggest that auricular acupuncture has beneficial effects in combatting the mechanisms of obesity [10], but there are only a limited number of evidence-based trials [11]. Recent systematic reviews and meta-analyses concerning obesity have focused on body acupuncture [12], acupoint catgut embedding [13], and pharmacoacupuncture [14]. There has been little comprehensive information published focusing on auricular acupoint stimulation in the treatment of obesity. The aim of this study is to perform an updated systematic review and meta-analysis to evaluate the effect of auricular acupoint stimulation, including auricular acupuncture and auricular acupressure, on overweight and obese adults using anthropometric measurements.

## 2. Methods

This systematic review and meta-analysis were conducted in accordance with the PRISMA-P guidelines (Appendix S1).

### 2.1. Data Sources and Search Strategy

We searched the following databases from inception to April 2017: PubMed, EMBASE, Allied and Complementary Medicine Database (AMED), China National Knowledge Infrastructure (CNKI), and the PerioPath Index to Taiwan Periodical Literature. We used the keywords: “((Acupuncture, Ear) OR (auricular acupuncture) OR (auricular acupressure) OR (auricular acupoint stimulation) OR (auricular therapy) OR (moxibustion)) AND ((weight reduction) OR (overweight) OR (obesity)).” Of these, “Acupuncture, Ear” and “obesity” are Medical Subject Headings Terms. We did not limit the language, year, or article type to enable a comprehensive and thorough search. We also did not restrict the search to humans or adults. Tzu-Lin Yeh and Shu-Jung Liu each conducted the search independently. Disagreements were resolved through discussion with the third author, Tsung-Ping Pai. The search strategy employed is available in Appendix S2.

### 2.2. Study Selection and Methodological Quality Assessment

We included all publications fitting the purposes of our study that followed our inclusion eligibility criteria: (1) randomized controlled trials (RCTs) focused on overweight and obese human adults; (2) no effective treatment in the control arm besides lifestyle modification; (3) the acupoints in the intervention group that were restricted to the ears; and (4) outcome measurements that include at least one anthropometric measurement, either as a primary or as secondary outcome of the paper. We excluded articles that were (1) irrelevant to the topic or target population; (2) duplicate publications; (3) trials of a cross-over study; or (4) confounded by other factors like effective body acupuncture, medication, or other traditional Chinese medicine in the intervention or control arm.

**Figure 1:**
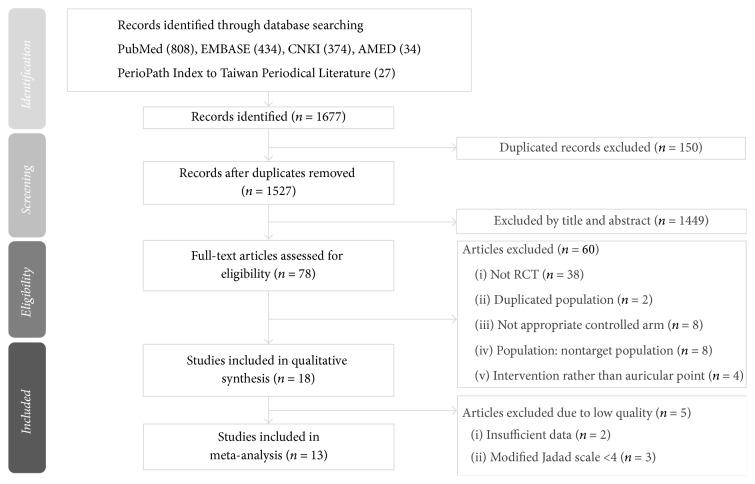
Flowchart of the trial selection process.

Authors Tzu-Lin Yeh and Hsin-Hao Chen independently used the modified Jadad scale to assess the methodological quality of each included study. The modified Jadad scale includes eight items to evaluate randomization, blinding, withdrawals, dropouts, inclusion and exclusion criteria, adverse reactions, and statistical analysis [15]. The score of each study ranges from zero (the lowest quality) to eight (the highest quality). Studies were classified as good to excellent (high quality) if they had a score of four or more. The detailed scores of each study are summarized in Appendix S3. If the two authors had different opinions when assessing and selecting the studies to include, agreement was reached by consensus with the third author, Tsung-Ping Pai. The study flow diagram is shown in Figure 1.

### 2.3. Data Extraction and Analysis

Tzu-Lin Yeh and Tsung-Ping Pai independently extracted the data from all included studies, and the following data were collected: first author's name, year of publication, study size, population characteristics, mean age, sex ratio, type of acupuncture, acupoint selection, treatment frequency, treatment duration, control group method, clinical outcome measurements, and adverse effects (Table 1).

Data were analyzed using the mean difference (MD) with 95% confidence intervals (CI) for continuous outcomes. RevMan version 5.3 software (Cochrane Collaboration) was used for all data analyses. The meta-analysis was conducted when the trials had acceptable clinical homogeneity and statistical heterogeneity. A random effect model was employed using DerSimonian and Laird's method due to the significant heterogeneity expected among the studies [16]. Heterogeneity was quantified using the Cochran *Q* test and *I*^2^ statistics [17, 18], and subgroup analyses were performed for different study settings. Metaregression was also performed using Comprehensive Meta-Analysis ver. 3 software (Biostat Inc., Englewood, NJ, USA) to explore the possible sources of heterogeneity. Potential publication bias was analyzed with a funnel plot and Egger's test [19].

## 3. Results

### 3.1. Study Characteristics

Figure 1 illustrates the search process and outcomes. A total of 18 RCTs were included for systematic review [20–37]. We assessed their quality using the modified Jadad scale. Characteristics of the included trials and total modified Jadad scores are shown in Table 1. Two RCTs [24, 25] had the same study population group, and the duplicated population was excluded when we reported the overall population size. We included both articles in our meta-analysis because they had different outcome measurements and did not interfere with our statistics.

All of the RCTs were published between 1995 and 2016. A total of 1775 participants were included, with a female to male ratio of 3 : 1, and a mean age of 38.9 years old. Six studies were conducted in Taiwan [23–25, 28, 31, 36], three studies each in China [26, 30, 37] and Iran [27, 29, 32], two studies in South Korea [33, 35], and one RCT in the USA [20], Egypt [21], Australia [22], and Austria [34].

### 3.2. Intervention

The intervention methods varied among the studies included. One trial in 1998 used an AcuSlim acupuncture device with electrical stimulation to the auricular acupoint [22]. Six RCTs [21, 23, 26, 31, 34, 35, 37] used traditional auricular acupuncture with stainless steel needles, and two of these also used electrical stimulation [21, 34]. Most of the included articles performed auricular acupressure with other devices such as* Sinapis alba* seeds [33], metal beads [31],* Vaccaria* seeds [25, 27–30, 32, 36, 37], an Acu-Stop 2000 device [20], or a Japanese Magnetic Pearl [25]. One of these RCTs also used electrical stimulation [36]. One trial [24] compared two different auricular acupressure devices (Japanese Magnetic Pearl and* Vaccaria* seeds) with a placebo. The number of auricular acupoints varied from one to six, with an average of 3.9. The most commonly used acupoints were Shen Men (TF4) and Stomach (CO4). Treatment length ranged from 3 to 12 weeks, with an average of 6.9 weeks.

### 3.3. Controls

Various sham acupuncture methods were used in the control arms of our included studies. No intervention was mentioned in two trials [30, 33]. One study in 1995 [20] and one trial in 1998 [22] used irrelevant acupuncture points in the wrist and thumb. Four articles used nonacupoints [21, 26, 36, 37]. Several studies used placebo needles or pseudo-interventions, including surgical tape [24, 25, 28], needles without needle points [23, 31], acupressure devices without seeds [27, 29], or electric stimulation with no power supply [34]. One study used superficial needling of the same points used in the treatment arm by removing the needles immediately after insertion [35].

### 3.4. Outcome Measures

Every RCT enrolled in this study included anthropometric measurements. In addition, seven trials [23, 24, 26, 27, 31, 36, 37] investigated biochemical characteristics and five studies [23, 26, 29, 31, 36] evaluated obesity-related hormone peptides. Psychological factors were evaluated using self-administered questionnaires in two studies [31, 33]. One publication [22] investigated appetite changes. Our objective was to ascertain the effect of auricular acupoint stimulation on anthropometric measurements, specifically.

### 3.5. Results of Meta-Analysis

Two studies [34, 36] did not have sufficient data to perform a meta-analysis. We contacted the authors, but there was no raw data available. Three articles had a relatively low Jadad score of 3 and thus were excluded from our meta-analysis [21, 26, 37]. Eventually, thirteen RCTs that achieved a modified Jadad score greater than or equal to four were included in our meta-analysis. The meta-analysis results are shown in Table 2.

**Figure 2:**
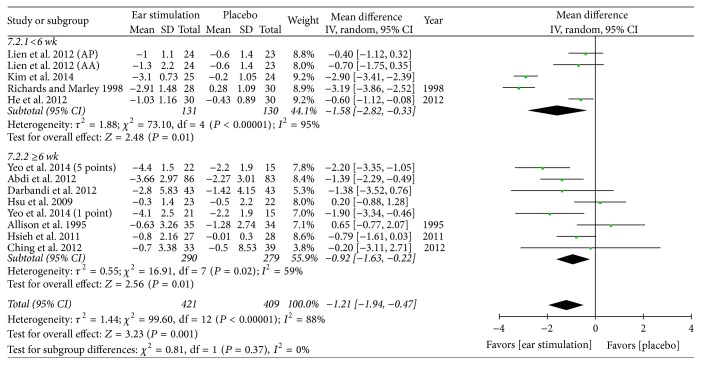
The forest plot of outcome measure “body weight change magnitude.”* Comparison*. Auricular stimulation versus placebo. Subgroup analysis by treatment duration: shorter or longer or equal to six weeks.

**Figure 3:**
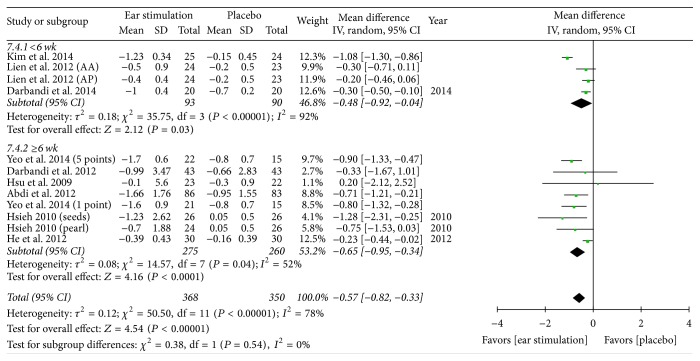
The forest plot of outcome measure “body mass index change magnitude.”* Comparison*. Auricular stimulation versus placebo. Subgroup analysis by treatment duration: shorter or longer or equal to six weeks.

**Figure 4:**
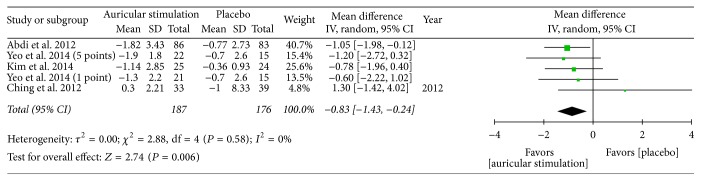
The forest plot of outcome measure “body fat change magnitude.”* Comparison*. Auricular stimulation versus placebo.

**Figure 5:**
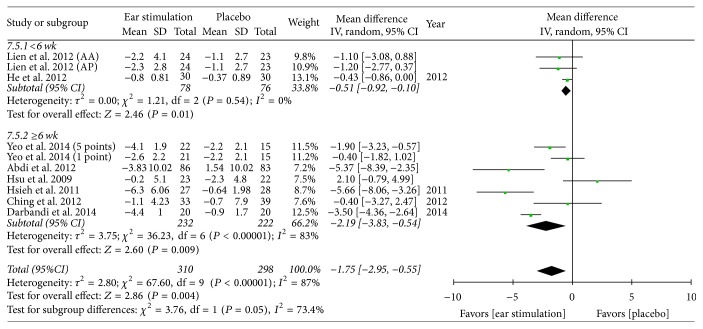
The forest plot of outcome measure “waist circumference change magnitude.”* Comparison*. Auricular stimulation versus placebo. Subgroup analysis by treatment duration: shorter or longer or equal to six weeks.

**Figure 6:**
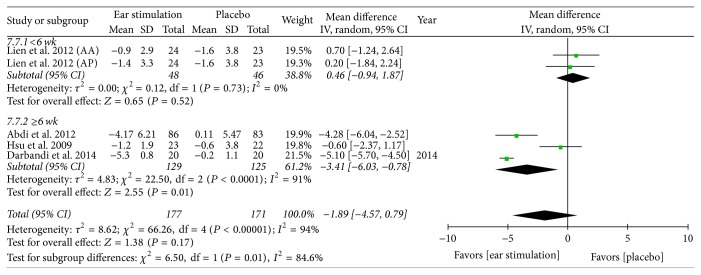
The forest plot of outcome measure “hip circumference change magnitude.”* Comparison*. Auricular stimulation versus placebo. Subgroup analysis by treatment duration: shorter or longer or equal to six weeks.

**Figure 7:**
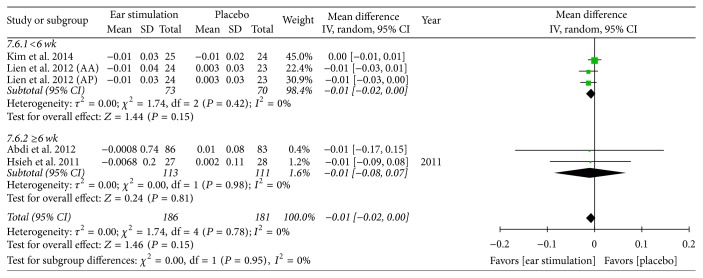
The forest plot of outcome measure “waist-to-hip ratio change magnitude.”* Comparison*. Auricular stimulation versus placebo. Subgroup analysis by treatment duration: shorter or longer or equal to six weeks.

Overall, the meta-analysis showed a significant reduction in BW, with an MD of −1.21 kg, a 95% CI of −1.94 to −0.47, *P* = 0.001, and *I*^2^ = 88% (forest plots are shown in Figure 2). The meta-analysis also showed a significant reduction in BMI (MD: −0.57 kg/m^2^; 95% CI: −0.82 to −0.33; *I*^2^ = 78%; *P* < 0.001; Figure 3), BF (MD: −0.83%; 95% CI: −1.43 to −0.24; *P* = 0.006; *I*^2^ = 0%; Figure 4), and WC (MD: −1.75 cm; 95% CI: −2.95 to −0.55; *P* = 0.004; *I*^2^ = 87%; Figure 5). No significant change was noted in HC (MD: −1.89 cm; 95% CI: −4.57 to 0.79; Figure 6) or WHR (MD: −0.01; 95% CI: −0.02~0.00; Figure 7).

### 3.6. Results of the Subgroup Analysis

Across our included studies, the treatment length ranged from 3 to 12 weeks, with an average of 6.9 weeks. Due to relatively high heterogeneity, we performed a subgroup analysis according to the intervention duration of less than six weeks (shorter) versus more than or equal to six weeks (longer).

BW decreased in both the shorter (MD: −1.58 kg; 95% CI: −2.82 to −0.33; *P* = 0.01; *I*^2^ = 95%; Figure 2) and the longer (MD: −0.92 kg; 95% CI: −1.63 to −0.22; *P* = 0.01; *I*^2^ = 59%; Figure 2) treatment subgroups. Both the shorter (MD: −0.48 kg/m^2^; 95% CI: −0.92 to −0.04; *P* = 0.03; *I*^2^ = 92%; Figure 3) and longer (MD: −0.65 kg/m^2^; 95% CI: −0.95 to −0.34; *P* < 0.001; *I*^2^ = 52%; Figure 3) treatment subgroups also showed a decreased BMI. Beneficial changes in WC were noted in the shorter subgroup (MD: −0.51 cm; 95% CI: −0.92 to −0.10; *P* = 0.01; *I*^2^ = 0%; Figure 5) and in the longer subgroup (MD: −2.19 cm; 95% CI: −3.83 to −0.54; *P* = 0.009; *I*^2^ = 83%; Figure 5). No positive effect on HC was observed in the shorter subgroup (MD: 0.46 cm: 95% CI: −0.94 to 1.87; Figure 6). However, HC significantly decreased in the longer treatment subgroup (MD: −3.41 cm; 95% CI: −6.03 to −0.78; *P* = 0.01; *I*^2^ = 91%). There was no significant reduction in WHR in either the shorter (MD: −0.01; 95% CI: −0.02 to 0.00; Figure 7) or longer (MD: −0.01; 95% CI: −0.08 to 0.07; Figure 7) treatment subgroups.

### 3.7. Results of the Metaregression

**Figure 8:**
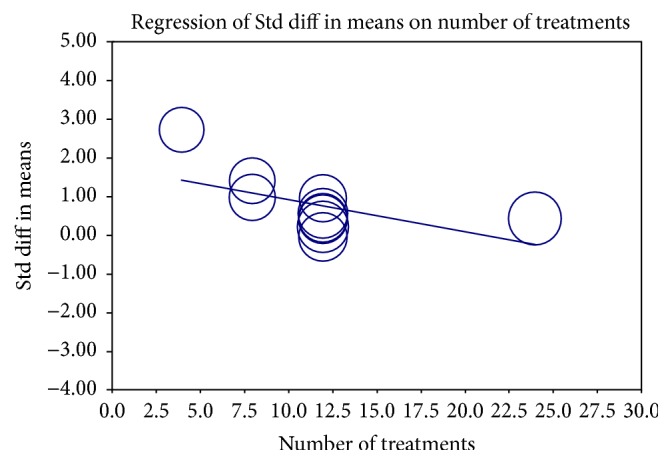
Regression of numbers of auricular stimulation treatment on body weight. Std diff, standard difference.

We further conducted a metaregression to explore potential interstudy heterogeneity in some of the pooled analyses. Participant characteristics such as sex and age; characteristics of the included publications such as modified Jadad score and dropout rate; and treatment differences such as numbers of acupoints and the frequency of auricular stimulation were analyzed. Total numbers of treatment were the only possible source of heterogeneity in BMI, with a −0.083 kg/m^2^ loss for every auricular stimulation (*P* = 0.036) (Figure 8).

### 3.8. Publication Bias

A funnel plot and Egger's test were used for the evaluation of potential publication bias. For BW and BMI, the* P* value of Egger's test was lower than 0.05, indicating that publication bias may exist. For BF, HC, WC, and WHR, there was no evidence of significant publication bias in our meta-analysis (*P* > 0.05, Appendix S4).

## 4. Discussion

In this study, we focused on the effects of auricular acupoint stimulation in overweight and obese adults. We systematically reviewed RCTs with a low risk of bias. Only RCTs achieving a moderate to high quality score (greater than or equal to 4) using the modified Jadad scale were included in our meta-analysis. Results of the meta-analysis suggest that, in overweight and obese adults with moderate to high heterogeneity, auricular acupoint stimulation had significant beneficial effects on the anthropometric parameters of BW, BMI, BF, and WC, while it showed less of an effect on HC and WHR. Heterogeneity improved after performing the subgroup analysis for the different treatment durations. After performing the subgroup analysis, only auricular stimulation of longer than 6 weeks produced a favorable effect on HC. Compared to shorter treatment durations, longer treatments of more than six weeks resulted in further decrease of BMI, WC, and HC. A linear effect was noted on BMI as the number of auricular stimulations increased.

A number of complementary therapies have been proposed in the treatment of overweight and obese individuals. A previous review found that acupuncture had some beneficial effects on obesity compared to a placebo or lifestyle control [10]. However, these results are of limited value due to the poor methodological quality of the included studies [38]. Another previous systematic review in 2012 focused on Chinese medicine and acupuncture in the treatment of obesity and reported that acupuncture was more effective than a placebo or lifestyle modification in reducing BW [39]. A total of 47 studies were included to evaluate the effects of acupuncture on obesity, but most of the included studies had a relatively low Jadad score of 1 to 3 points. Only one study, in 1998, had a Jadad score of 5, and this study was included in the present systematic review [22].

A recent meta-analysis performed in 2017 also indicated that acupuncture is an effective treatment for obesity [12]. However, the article emphasized body acupuncture, and BMI was the only reported outcome. Furthermore, twelve out of the 21 studies included had unclear randomization and only two studies achieved a score of 4 on the Jadad scale. An asymmetric funnel plot indicates the potential for publication bias.

Our main findings are consistent with an earlier systematic review and meta-analysis that focused on auricular acupuncture [40]. The article concluded that the effect of auricular acupuncture in combination with diet and exercise was more effective than auricular acupuncture alone. We used all of the articles included, except one [41], in the present review. The target population of the previous review was obese and nonobese adults, which was incongruent with our study's purposes. Furthermore, the authors did not perform a subgroup analysis, and all of their interventions were defined as auricular acupuncture. We considered several of these RCTs [20, 25, 27, 29, 30] as auricular acupressure in our review and subgroup analysis. Furthermore, in the previous review, only five articles were included in the meta-analysis and BW was the only reported outcome.

In the present review, our aim was to provide new evidence for auricular acupoint stimulation and to report comprehensive anthropometric parameters. Our strategy has several strengths. First, all of our included publications used a randomized placebo-controlled design, which reduces the risk of bias. Second, all of the articles were critically appraised using the modified Jadad scale and had a relatively high score of greater than or equal to four. Third, to the best of our knowledge, this systematic review included the first meta-analysis focused on auricular acupoint stimulation in overweight and obese adults. Finally, we comprehensively report a number of anthropometric parameters and how they were affected.

There are also several limitations to this study. First, most of the participants in our included studies were middle-aged Asian women. Our conclusions may be appropriate for this population, but the current evidence may not provide a strong case in other populations. Second, there was substantial heterogeneity. We explored the possible sources of this heterogeneity by conducting a subgroup analysis. The significant variations in acupoint selection, type of auricular therapy, treatment duration, and study endpoints are likely responsible for the heterogeneity in our meta-analysis.

Auricular stimulation may be involved in several mechanisms of BW regulation and obesity such as anorexigenic and orexigenic peptides, glucose metabolism, insulin resistance, lipid metabolism, and inflammatory markers [10]. Part of the cavum conchae is innervated by the auricular branch of the vagal nerve [42] which is stimulated in order to achieve a degree of appetite suppression [22]. Stimulation of cholinergic nerves may reduce plasma glucose levels [43] and improve insulin resistance [44] through serotonin-induced secretion of *β* endorphin from the adrenal gland [45] and insulin growth factor-1 [10]. It also suppresses the innate inflammatory response via the acetylcholine-induced suppression of cytokine synthesis [46]. In an animal study, activation of this pathway significantly improved glucose homeostasis and insulin sensitivity via the suppression of adipose tissue inflammation without changes in body weight in both genetically obese and diet-induced obese mice [47].

Traditionally, auricular acupuncture includes needle insertion with or without the application of electrical stimulation to ear acupoints [48]. In contrast, acupressure does not involve needles and does not usually result in strong painful sensations. Acupressure often involves using various plant seeds or magnetic pellets taped onto both ears to stimulate acupoints. The various types of acupressure may explain why heterogeneity improved in the acupuncture subgroup but was still present in the acupressure subgroup. Acupressure is relatively noninvasive, low-cost, and self-managed. Once the seeds have been applied, they can remain on the ears for up to one month, depending on skin condition. Patients can stimulate these acupoints by pressing them with fingers as directed to achieve acupuncture-like effects.

Although the effect of anthropometric parameters changes was not large, studies have reported health benefits with a weight loss of only 3~5 percent of BW and complications of obesity could be reversed [6, 49]. In our study, longer treatments had a more favorable effect in BMI, WC, and HC than shorter treatment durations. The more the number of treatments is, the more the BMI decreased. Therapeutic lifestyle modification along with more auricular acupoint stimulations with longer treatment durations may be a choice for obese and overweight adults.

The selection of acupoints is key for treatment success [50]. Only a few qualified studies have discussed treatment outcome differences depending on acupoint selection. The number and location of acupuncture points varied in the RCTs included in the present study. Ear charts vary in somatotopic arrangement, so it is necessary for therapists to gather more data to form recommendations for an international standard of auricular acupoints [51]. Treatment duration is another important factor affecting outcome. In our meta-analysis, treatment duration was short and treatment frequency varied. It has been reported that two to ten weeks of auricular therapy provides treatment benefits [52], although evidence for this is still insufficient. In addition, sham intervention designs have yet to be standardized. Sham acupuncture methods can be broadly categorized into five approaches [53, 54]: superficial needling of the same points used in the treatment arm; needling of irrelevant acupuncture points; needling nonacupoints; using placebo needles; and employing pseudo-interventions. Unlike body acupuncture, it is more difficult to locate nonacupoints in the ear for certain sham interventions due to the small size of the ear and large number of identified acupoints. There have been no solid conclusions concerning which design is the most appropriate to use in a control group [53], further increasing the heterogeneity between studies. Future studies should focus on larger populations, emphasize standardized auricular acupoint stimulation, and use standard sham methods with a modest treatment duration and frequency to ensure that eligible RCTs provide good quality evidence.

## 5. Conclusion

This meta-analysis shows that auricular acupoint stimulation improves physical anthropometric parameters including BW, BMI, BF, and WC in overweight and obese adults. These treatments have less of an effect on HC and WHR. But auricular stimulation longer than 6 weeks had a favorable effect on HC after performing a subgroup analysis. A linear effect was noted on BMI as the number of auricular stimulations increased. Therefore, we recommend more auricular acupoint stimulations of longer than 6 weeks as an alternative treatment for overweight and obese adults.

## Figures and Tables

**Table 1:** Characteristics of the included studies.

Study (year)	Country	Population	Modified Jadad score	Sample size	Characteristics of participants (gender, age)	Intervention group treatment	Control group treatment	Frequency/treatment course	Main outcome	Adverse effect
Allison et al. (1995) [20]	USA	Obese adults	5	96	16 M/80 F 41.1	AA device (Acu-Stop 2000) on dominant ear, massaged, 4 times a day	Subjects received wrist acupressure device in dominant side massaged, 4 times a day	Every 2 wks/12 wks	(1) BW, BF, BFM (2) BP (3) Compliance	3 bleeding ears in treatment group

Shafshak (1995) [21]	Egypt	Obese female adults	3	30	0 M/30 F 21–35	Stainless needle AA with ES to AP1 or AP2. ES duration = 25 mins AP1: CO4 AP2: HG	Stainless needle AA with ES to sham AP on ear ES duration = 25 mins	Every day/3 wks	BW	NR

Richards and Marley (1998) [22]	Australia	Overweight adults	7	60	NR >18	ES with AcuSlim device to AP ES duration = 15–20 mins AP: CO4, TF4	ES with AcuSlim device to thumb (without AP) ES duration = 15–20 mins	Twice daily/4 wks	(1) BW^*∗*^ (2) Appetite change^*∗*^	NR

Hsu et al. (2009) [23]	Taiwan	Obese female adults	7.5	45	0 M/45 F 39.5 ± 12.0	Stainless steel needle (0.2 cm needle point) to AP; no apply any pressure to AP AP: CO4, CO18, HG, TF4	Sham AA using placebo needles (without needle point) AP: CO4, CO18, HG, TF4	Twice a wk/6 wks	(1) BW, BMI, WC, HC (2) FBG, TG, TCH, HDL-C, LDL-C (3) Insulin, leptin, adiponectin, ghrelin, HOMA-IR	1 mild inflammation; 9 mild tenderness cases

Hsieh (2010) [24]	Taiwan	Overweight adolescents	4	84	8 M/76 F 18–20	Auricular acupressure to Japanese Magnetic Pearl or *Vaccaria* seeds	Acupressure tape (0.5*∗*0.5 cm) on ear	NR/8 wks	(1) BMI (2) TCH, TG, HDL-C, LDL-C	NR

Hsieh et al. (2011) [26]	China	Overweight adults	3	200	NR 18–65	*Vaccaria* seeds to AP; applied pressure to each AP for 30–50 times before eating AP: CO4, CO17, CO18, TF4	Needle AA on sham AP on the body	Every two days/12 wks	(1) BW, BMI, BF (2) TCH, TG (3) FINS, HOMA-IR	NR

Hsieh et al. (2011) [25]	Taiwan	Young adults with abdominal obesity	4	55	5 M/50 F 18–20	Acupressure with Japanese Magnetic Pearl on AP AP: CO1, CO4, CO6, CO18, TF4	Adhesive tape to AP AP: CO1, CO4, CO6, CO18, TF4	NR/8 wks	BW, WC	NR

Abdi et al. (2012) [27]	Iran	Overweight adults	4	169	NR 38	Ear pressing plaster with seed to AP Apply pressure to AP before eating for 20 sec AP: CO1, CO4, CO17, HG, HX1, TF4	Ear pressing plaster without seed to sham AP AP: AH5, CO2, CO13	Twice a wk/12 wks	(1) BW^*∗*^, BMI^*∗*^, BF^*∗*^, WC^*∗*^, HC^*∗*^, WHR (2) FBG, TCH, TG, HDL-C, LDL-C, hs-CRP (3) Anti-HSP27^*∗*^, Anti-HSP60^*∗*^, Anti-HSP65^*∗*^, anti-HSP70^*∗*^	No adverse effect

Ching et al. (2012) [28]	Taiwan	Overweight schizophrenia patients	7.5	72	33 M/39 F 47.8	*Vaccaria* seeds to AP. Apply pressure to AP before eating Each AP for 1 min AP: CO4, CO18, HG, TF4	Surgical tape to AP. AP points were not pressed AP: CO4, CO18, HG, TF4	Twice a wk/8 wks	BW, BMI, BF	NR

Darbandi et al. (2012) [29]	Iran	Overweight adults	6	86	12 M/74 F 37.7 ± 9.5	*Vaccaria* seeds to AP. Apply pressure to AP before eating AP: CO1, CO4, CO17, HG, HX1, TF4	Ear plaster without seeds to AP AP: AH5, CO2, CO13, Nose	Twice a wk/6 wks	(1) BW, BMI, BFM (2) Leptin^*∗*^	No adverse effect

He et al. (2012) [30]	China	Obese female adults	4.5	60	0 M/60 F 34	*Vaccaria* seeds to AP. Apply pressure on each seed for 30 secs/day AP: CO4, CO7, CO13, CO18, HG, TF4	No AA	Every three days/4 wks	BW, BMI, WC	NR

Lien et al. (2012) [31]	Taiwan	Obese female adults	7.5	71	0 M/71 F 40.7 ± 11.3	Apply stainless needles with a 0.2 cm tip or magnetic metal beads to AP AP: CO4, CO18, HG, TF4	Sham AA to AP with auricular needle with tips removed (needle without needle points) AP: CO4, CO18, HG, TF4	Three times a wk/4 wks	(1) BW, BMI, WC, HC, WHR (2) FBG, TG, TCH, HDL-C, LDL-C (3) Adiponectin, insulin, ghrelin, Leptin, HOMA-IR (4) WHO BREF life-quality scores	1 account of dizziness after AA

Darbandi et al. (2014) [32]	Iran	Obese male adults	7.5	40	40 M 38.5	*Vaccaria* seeds to AP on both ears for 3 days. Apply pressure to AP before eating AP: CO1, CO4, CO17, HG, HX1, TF4	Sham AA with plasters to AP on both ears for 3 days. Apply pressure to AP before eating AP: CO2, CO13, AH5	Twice a wk/6 wks	BW, BMI, BFM, HC	No adverse effect

Kim et al. (2014) [33]	South Korea	Obese female young adults	5	49	0 M/49 F 20.7 ± 1.1	Three *Sinapis* alba seeds to each AP. Apply pressure to AP for 5 secs *∗* 10 times/point, three courses/day AP: CO1, CO4, CO6, TF4	NO AA	Weekly/4 wks	(1) BW^*∗*^, BMI^*∗*^, BF, WHR (2) Self-efficacy scale^*∗*^	NR

Schukro et al. (2014) [34]	Austria	Obese female individuals	7	42	0 M/42 F	ES with a P-Stim device AP: CO4, CO7, HG	P-Stim® dummy (no power supply) AP: CO4, CO7, HG	Weekly/6 wks	BW^*∗*^, BMI^*∗*^, BF	NR

Yeo et al. (2014) [35]	South Korea	Overweight adults	6.5	58	6 M/52 F 38.6 ± 11.8	Acupuncture needle on AP1 or AP2 AP1: CO1, CO4, CO6, CO18, HX1; AP2: HG	Sham AA, removed immediately after insertion; AP: CO1, CO4, CO6, CO18, HX1	Weekly/8 wks	(1) BW^*∗*^, BMI^*∗*^, WC^*∗*^, BFM^*∗*^, BF (2) BP	NR

Yeh et al. (2015) [36]	Taiwan	Obese adults	7.5	70	35 M/35 F 31.3 ± 8.8	ES to AP and then apply pressure to each AP with *Vaccaria *seeds for 1 min *∗* 4 times/day AP: CO4, CO18, HG, TF4	ES to sham AP. Apply pressure to each AP with *Vaccaria* seeds for 1 min *∗* 4 times/day AP: AH3, SF3, SF4, SF6	Weekly/10 wks	(1) BMI (2) BP, TCH, TG (3) Adiponectin, leptin	NR

Hsu (2016) [37]	China	Overweight adults	3	120	73 M/47 F 38.7 ± 10.0	*Vaccaria* seeds to AP on each ear alternating every 2 days AP: CO17, CO18, HG, TF4	Needle AA to sham AP on body	Every 2 days/12 wks	(1) BW, BMI, BF (2) TCH, TG (3) FINS, HOMA-IR	NR

Values are expressed as the mean ± standard deviation. ^*∗*^*P* < 0.05 between treatment and control groups; AA: auricular acupuncture; anti-HSP: anti-heat shock protein; AP: auricular acupoint; BF: body fat percentage; BFM: body fat mass; BMI: body mass index; BP: blood pressure; BW: body weight; ES: electrical stimulation; F: female; FBG: fasting blood glucose; FINS: fasting insulin; HC: hip circumference; HDL-C: high-density lipoprotein cholesterol; HOMA-IR: homeostasis model assessment for insulin resistance; HR: heart rate; hs-CRP: high-sensitivity C-reactive protein; LDL-C: low-density lipoprotein cholesterol; M: male; min: minutes; NR: not reported; sec: second; TCH: total cholesterol; TG: triglycerides; WC: waist circumference; WHR: waist-to-hip ratio; wk: week; acupoints: ankle (AH3), hip (AH5), mouth (CO1), esophagus (CO2), stomach (CO4), small intestine (CO6), large intestine (CO7), spleen (CO13), San Jiao (CO17), endocrine (CO18), hunger point (HG), center of ear (HX1), elbow (SF3), shoulder (SF4), clavicle (SF6), Shen Men (TF4).

**Table 2:** The effect of auricular acupoint stimulation on anthropometric measurements.

Outcome	Intervention	Studies, *N*	Participants	MD (95% CI)	*P*	*I* ^2^
BW	Overall auricular stimulation	13	830	−1.21 (−1.94, −0.47)	0.001	88%
	<6 weeks' treatment	4	165	−1.58 (−2.82, −0.33)	0.01	95%
	≧6 weeks' treatment	9	665	−0.92 (−1.63, −0.22)	0.01	59%

BMI	Overall auricular stimulation	12	718	−0.57 (−0.82, −0.33)	<0.001	78%
	<6 weeks' treatment	4	165	−0.48 (−0.92, −0.04)	0.03	92%
	≧6 weeks' treatment	8	553	−0.65 (−0.95, −0.34)	<0.001	52%

BF	Overall auricular stimulation	5	363	−0.83 (−1.43, −0.24)	0.006	0%

WC	Overall auricular stimulation	10	608	−1.75 (−2.95, −0.55)	0.004	87%
	<6 weeks' treatment	4	165	−0.51 (−0.92, −0.10)	0.01	0%
	≧6 weeks' treatment	6	443	−2.19 (−3.83, −0.54)	0.009	83%

HC	Overall auricular stimulation	5	348	−1.89 (−4.57, 0.79)	0.17	94%
	<6 weeks' treatment	2	92	0.46 (−.0.94, 1.87)	0.52	0%
	≧6 weeks' treatment	3	256	−3.41 (−6.03, −0.78)	0.01	91%

WHR	Overall auricular stimulation	5	367	−0.01 (−0.02, 0.00)	0.15	0%
	<6 weeks' treatment	1	47	−0.01 (−0.02, 0.00)	0.15	0%
	≧6 weeks' treatment	4	320	−0.01 (−0.08, 0.07)	0.81	0%

BF: body fat; BMI: body mass index; BW: body weight; CI: confidence interval; HC: hip circumference; MD: mean difference; WC: waist circumference; WHR: waist-to-hip ratio.

## Supplementary Materials

Supplementary Materials Appendix S1, Table  1. PRISMA checklist. Appendix S2, Table  2. Search strategy. Appendix S3, Table  3. Detailed modified Jadad scale of each included studies. Appendix S4, Figure  1. The funnel plot and Egger's test to identify publication bias. Appendix S4, Figure  1. The funnel plot and Egger's test to identify publication bias. BW, body weight; BMI, body mass index; BF, body fat; WC, waist circumference; HC, hip circumference; WHR, waist-to-hip ratio.
