# Supplementary material for: The Effect of Auricular Acupoint Stimulation in Overweight and Obese Adults: A Systematic Review and Meta-Analysis of Randomized Controlled Trials
Source: Evid Based Complement Alternat Med. 2017 Dec 5;2017:3080547. doi: 10.1155/2017/3080547 (PMC5735786; doi:10.1155/2017/3080547)
Supplement: Supplementary Materials — Appendix S1, Table 1. PRISMA checklist. Appendix S2, Table 2. Search strategy. Appendix S3, Table 3. Detailed modified Jadad scale of each included studies. Appendix S4, Figure 1. The funnel plot and Egger's test to identify publication bias. Appendix S4, Figure 1. The funnel plot and Egger's test to identify publication bias. BW, body weight; BMI, body mass index; BF, body fat; WC, waist circumference; HC, hip circumference; WHR, waist-to-hip ratio. [file 3080547.f1.doc]

Appendix S1 TABLE 1: PRISMA checklist.

| **Section/topic** | **#** | **Checklist item** | **Reported on page #** |
| --- | --- | --- | --- |
| **TITLE** | | |  |
| Title | 1 | Identify the report as a systematic review, meta-analysis, or both. | 1 |
| **ABSTRACT** | | |  |
| Structured summary | 2 | Provide a structured summary including, as applicable: background; objectives; data sources; study eligibility criteria, participants, and interventions; study appraisal and synthesis methods; results; limitations; conclusions and implications of key findings; systematic review registration number. | 2 |
| **INTRODUCTION** | | |  |
| Rationale | 3 | Describe the rationale for the review in the context of what is already known. | 3 |
| Objectives | 4 | Provide an explicit statement of questions being addressed with reference to participants, interventions, comparisons, outcomes, and study design (PICOS). | 3 |
| **METHODS** | | |  |
| Protocol and registration | 5 | Indicate if a review protocol exists, if and where it can be accessed (e.g., Web address), and, if available, provide registration information including registration number. | N/A |
| Eligibility criteria | 6 | Specify study characteristics (e.g., PICOS, length of follow-up) and report characteristics (e.g., years considered, language, publication status) used as criteria for eligibility, giving rationale. | 4 |
| Information sources | 7 | Describe all information sources (e.g., databases with dates of coverage, contact with study authors to identify additional studies) in the search and date last searched. | 4 |
| Search | 8 | Present full electronic search strategy for at least one database, including any limits used, such that it could be repeated. | 4, Appendix S2 |
| Study selection | 9 | State the process for selecting studies (i.e., screening, eligibility, included in systematic review, and, if applicable, included in the meta-analysis). | 4-5, Figure 1 |
| Data collection process | 10 | Describe method of data extraction from reports (e.g., piloted forms, independently, in duplicate) and any processes for obtaining and confirming data from investigators. | 4-5 |
| Data items | 11 | List and define all variables for which data were sought (e.g., PICOS, funding sources) and any assumptions and simplifications made. | 4-5 |
| Risk of bias in individual studies | 12 | Describe methods used for assessing risk of bias of individual studies (including specification of whether this was done at the study or outcome level), and how this information is to be used in any data synthesis. | 4 |
| Summary measures | 13 | State the principal summary measures (e.g., risk ratio, difference in means). | 5 |
| Synthesis of results | 14 | Describe the methods of handling data and combining results of studies, if done, including measures of consistency (e.g., I^2^) for each meta-analysis. | 5 |

Page 1 of 2

| **Section/topic** | **#** | **Checklist item** | **Reported on page #** |
| --- | --- | --- | --- |
| Risk of bias across studies | 15 | Specify any assessment of risk of bias that may affect the cumulative evidence (e.g., publication bias, selective reporting within studies). | 5 |
| Additional analyses | 16 | Describe methods of additional analyses (e.g., sensitivity or subgroup analyses, meta-regression), if done, indicating which were pre-specified. | 5 |
| **RESULTS** | | |  |
| Study selection | 17 | Give numbers of studies screened, assessed for eligibility, and included in the review, with reasons for exclusions at each stage, ideally with a flow diagram. | 6, Figure 1 |
| Study characteristics | 18 | For each study, present characteristics for which data were extracted (e.g., study size, PICOS, follow-up period) and provide the citations. | 6-7, Table 1 |
| Risk of bias within studies | 19 | Present data on risk of bias of each study and, if available, any outcome level assessment (see item 12). | 6, Appendix S3 |
| Results of individual studies | 20 | For all outcomes considered (benefits or harms), present, for each study: (a) simple summary data for each intervention group (b) effect estimates and confidence intervals, ideally with a forest plot. | 7, Figure 2~7 |
| Synthesis of results | 21 | Present results of each meta-analysis done, including confidence intervals and measures of consistency. | 7 |
| Risk of bias across studies | 22 | Present results of any assessment of risk of bias across studies (see Item 15). | 8, Appendix S4 |
| Additional analysis | 23 | Give results of additional analyses, if done (e.g., sensitivity or subgroup analyses, meta-regression [see Item 16]). | 7-8 |
| **DISCUSSION** | | |  |
| Summary of evidence | 24 | Summarize the main findings including the strength of evidence for each main outcome; consider their relevance to key groups (e.g., healthcare providers, users, and policy makers). | 9 |
| Limitations | 25 | Discuss limitations at study and outcome level (e.g., risk of bias), and at review-level (e.g., incomplete retrieval of identified research, reporting bias). | 10 |
| Conclusions | 26 | Provide a general interpretation of the results in the context of other evidence, and implications for future research. | 12 |
| **FUNDING** | | |  |
| Funding | 27 | Describe sources of funding for the systematic review and other support (e.g., supply of data); role of funders for the systematic review. | 12, N/A |

*From:*  Moher D, Liberati A, Tetzlaff J, Altman DG, The PRISMA Group (2009). Preferred Reporting Items for Systematic Reviews and Meta-Analyses: The PRISMA Statement. PLoS Med 6(7): e1000097. doi:10.1371/journal.pmed1000097

For more information, visit: **www.prisma-statement.org**.

Page 2 of 2

Appendix S2 TABLE 2: Search strategy.

PubMed

| **Search** | **Query** |
| --- | --- |
| **#1** | Search Acupuncture, Ear |
| **#2** | Search Auriculotherapy |
| **#3** | Search Auricular Acupuncture* |
| **#4** | Search Auriculotherap* |
| **#5** | Search (((Acupuncture, Ear) OR Auriculotherapy) OR Auricular Acupuncture*) OR Auriculotherap* |
| **#6** | Search acupuncture OR Acupuncture Therapy OR pharmacoacupuncture OR Acupuncture Point OR Acupoint* OR acupressure |
| **#7** | Search Shiatsu[Title/Abstract] OR Zhi Ya[Title/Abstract] OR Chih Ya[Title/Abstract] OR Shiatzu[Title/Abstract] |
| **#8** | Search moxibustion OR Moxabustion OR mugwort OR moxa OR Meridians OR Jingluo OR stimulation OR Physical Stimulation |
| **#9** | Search needle stick* |
| **#10** | Search ((((((((Acupuncture, Ear) OR Auriculotherapy) OR Auricular Acupuncture*) OR Auriculotherap*)) OR (acupuncture OR Acupuncture Therapy OR pharmacoacupuncture OR Acupuncture Point OR Acupoint* OR acupressure)) OR (Shiatsu[Title/Abstract] OR Zhi Ya[Title/Abstract] OR Chih Ya[Title/Abstract] OR Shiatzu[Title/Abstract])) OR (moxibustion OR Moxabustion OR mugwort OR moxa OR Meridians OR Jingluo OR stimulation OR Physical Stimulation)) OR needle stick* |
| **#11** | Search (ear OR auricular OR auric* OR ear point*) |
| **#12** | Search ((((((((((Acupuncture, Ear) OR Auriculotherapy) OR Auricular Acupuncture*) OR Auriculotherap*)) OR (acupuncture OR Acupuncture Therapy OR pharmacoacupuncture OR Acupuncture Point OR Acupoint* OR acupressure)) OR (Shiatsu[Title/Abstract] OR Zhi Ya[Title/Abstract] OR Chih Ya[Title/Abstract] OR Shiatzu[Title/Abstract])) OR (moxibustion OR Moxabustion OR mugwort OR moxa OR Meridians OR Jingluo OR stimulation OR Physical Stimulation)) OR needle stick*)) AND ((ear OR auricular OR auric* OR ear point*)) |
| **#13** | Search weight loss OR Weight Gain OR Body Weight Changes OR Overweight OR obesity OR Overnutrition OR Body Weight OR Weight Reduction Programs OR Ideal Body Weight OR Weight Loss* OR Weight Reduc* OR weight decre* OR weight increase OR weight control OR weight management OR BMI OR OR fat OR obese OR weight |
| **#14** | Search (Quetelet* AND Index) |
| **#15** | Search (Weights and Measures) |
| **#16** | Search (((weight loss OR Weight Gain OR Body Weight Changes OR Overweight OR obesity OR Overnutrition OR Body Weight OR Weight Reduction Programs OR Ideal Body Weight OR Weight Loss* OR Weight Reduc* OR weight decre* OR weight increase OR weight control OR weight management OR BMI OR OR fat OR obese OR weight)) OR ((Quetelet* AND Index))) OR ((Weights and Measures)) |
| **#17** | Search ((((((((((((Acupuncture, Ear) OR Auriculotherapy) OR Auricular Acupuncture*) OR Auriculotherap*)) OR (acupuncture OR Acupuncture Therapy OR pharmacoacupuncture OR Acupuncture Point OR Acupoint* OR acupressure)) OR (Shiatsu[Title/Abstract] OR Zhi Ya[Title/Abstract] OR Chih Ya[Title/Abstract] OR Shiatzu[Title/Abstract])) OR (moxibustion OR Moxabustion OR mugwort OR moxa OR Meridians OR Jingluo OR stimulation OR Physical Stimulation)) OR needle stick*)) AND ((ear OR auricular OR auric* OR ear point*)))) AND ((((weight loss OR Weight Gain OR Body Weight Changes OR Overweight OR obesity OR Overnutrition OR Body Weight OR Weight Reduction Programs OR Ideal Body Weight OR Weight Loss* OR Weight Reduc* OR weight decre* OR weight increase OR weight control OR weight management OR BMI OR OR fat OR obese OR weight)) OR ((Quetelet* AND Index))) OR ((Weights and Measures))) |

EMBASE

| **Collapsev** | |
| --- | --- |
| **#1** | 'auricular acupuncture' |
| **#2** | auriculotherap* |
| **#4** | auriculotherapy |
| **#6** | 'acupuncture' |
| **#7** | 'electroacupuncture' |
| **#8** | 'acupressure' |
| **#9** | 'moxibustion' |
| **#10** | moxabustion |
| **#11** | mugwort |
| **#12** | 'moxa' |
| **#13** | meridians |
| **#14** | jingluo |
| **#15** | 'stimulation' |
| **#16** | needle?AND?stick* |
| **#17** | #6 OR #7 OR #8 OR #9 OR #10 OR #11 OR #12 OR #13 OR #14 OR #15 OR #16 |
| **#18** | 'ear' |
| **#19** | auricular |
| **#20** | 'auricular acupressure' |
| **#21** | auric* |
| **#22** | #1 OR #2 OR #4 OR #20 |
| **#23** | #6 OR #7 OR #8 OR #9 OR #10 OR #11 OR #12 OR #13 OR #14 OR #15 OR #16 |
| **#24** | #18 OR #19 OR #21 |
| **#25** | #23 AND #24 |
| **#26** | #22 OR #25 |
| **#27** | 'body weight' |
| **#28** | 'weight reduction' |
| **#29** | 'weight gain' |
| **#30** | 'obesity' |
| **#31** | 'weight' |
| **#32** | 'fat' |
| **#33** | 'body mass' |
| **#34** | 'bmi' |
| **#35** | overweight |
| **#36** | obese |
| **#37** | quetelet*?AND?index |
| **#38** | 'overnutrition' |
| **#39** | #27 OR #28 OR #29 OR #30 OR #31 OR #32 OR #33 OR #34 OR #35 OR #36 OR #37 OR #38 |
| **#40** | #26 AND #39 |
| **#41** | acupoint* |
| **#42** | acupressure |
| **#43** | #17 OR #41 OR #42 |
| **#44** | ear |
| **#45** | #24 OR #44 |
| **#46** | #43 AND #45 |
| **#47** | weight |
| **#48** | #39 OR #47 |
| **#49** | #46 AND #48 |

Allied and Complementary Medicine Database (AMED)

| **1** | exp Ear acupuncture/ or Acupuncture, Ear.mp. |
| --- | --- |
| **2** | Auriculotherapy.mp. |
| **3** | (Auricular Acupuncture* or Auriculotherap*).mp. [mp=abstract, heading words, title] |
| **4** | 1 or 2 or 3 |
| **5** | acupuncture.mp. or exp Acupuncture/ |
| **6** | Acupuncture Therapy.mp. or exp Acupuncture therapy/ |
| **7** | Pharmacoacupuncture.mp. |
| **8** | exp Acupoints/ or Acupuncture Point.mp. |
| **9** | exp Acupressure/ or acupressure.mp. |
| **10** | exp Electroacupuncture/ or Electroacupuncture.mp. |
| **11** | exp Moxibustion/ or moxibustion.mp. |
| **12** | moxabustion.mp. [mp=abstract, heading words, title] |
| **13** | MUGWORT.mp. |
| **14** | exp Meridians/ or Meridians.mp. |
| **15** | Jingluo.mp. |
| **16** | Physical Stimulation.mp. or exp Physical stimulation/ |
| **17** | exp Electric stimulation/ or stimulation.mp. |
| **18** | needle stick*.mp. |
| **19** | 5 or 6 or 7 or 8 or 9 or 10 or 11 or 12 or 13 or 14 or 15 or 16 or 17 or 18 |
| **20** | exp Ear/ or ear.mp. |
| **21** | auricular.mp. |
| **22** | auric*.mp. [mp=abstract, heading words, title] |
| **23** | 20 or 21 or 22 |
| **24** | 19 and 23 |
| **25** | 3 or 24 |
| **26** | exp Obesity/ or exp Weight loss/ or weight loss.mp. |
| **27** | exp Body weight/ or Weight Gain.mp. |
| **28** | exp body mass index/ or Body Mass Index.mp. |
| **29** | Overweight.mp. |
| **30** | (Weight Loss* Weight Reduc* or weight decre* or weight increase or weight control or weight management).mp. [mp=abstract, heading words, title] |
| **31** | (BMI or fat or obses or weight).mp. [mp=abstract, heading words, title] |
| **32** | 26 or 27 or 28 or 29 or 30 or 31 |
| **33** | 25 and 32 |

China National Knowledge Infrastructure (CNKI)

| **1** | (耳針 OR 針灸 OR 扎針 OR 針刺 OR 穴位按壓 OR 穴位按摩 OR 指壓 OR 艾灸 OR 灸) |
| --- | --- |
| **2** | (減重 OR 減肥 OR 瘦身 OR 體重 OR BMI OR 體脂肪 OR 過重 OR 肥胖) |
| **3** | (耳) |
| **4** | 1 and 2 and 3 |

Appendix S3 TABLE 3: Detailed modified Jadad scale of each included studies.

| Included  studies | Randomized^a^ | Randomized appropriate^b^ | Blind^c^ | Blind appropriate^d^ | Withdraw dropout^e^ | Inclusion exclusion^f^ | Adverse effect^g^ | Statistical method^h^ | Total |
| --- | --- | --- | --- | --- | --- | --- | --- | --- | --- |
|
| Allison et al. (1995) | 1 | 0 | 0 | 0 | 1 | 1 | 1 | 1 | 5 |
| Shafshak et al. (1995) | 1 | 0 | 0 | 0 | 0 | 1 | 0 | 1 | 3 |
| Richards et al. (1998) | 1 | 1^i, j^ | 1 | 1^k^ | 1 | 1 | 0 | 1 | 7 |
| Hsu et al. (2009) | 1 | 1^l^ | 0.5 | 1^m^ | 1 | 1 | 1 | 1 | 7.5 |
| Hsieh et al. (2010) | 1 | 0 | 0 | 0 | 1 | 1 | 0 | 1 | 4 |
| Hsieh^1^ et al. (2011) | 1 | 0 | 0 | 0 | 0 | 1 | 0 | 1 | 3 |
| Hsieh^2^ et al. (2011) | 1 | 0 | 0 | 0 | 1 | 1 | 0 | 1 | 4 |
| Abdi et al. (2012) | 1 | 0 | 0 | 0 | 1 | 1 | 0 | 1 | 4 |
| Ching et al. (2012) | 1 | 1^j^ | 0.5 | 1^k^ | 1 | 1 | 1 | 1 | 7.5 |
| Darbandi et al. (2012) | 1 | 1^l^ | 0 | 0 | 1 | 1 | 1 | 1 | 6 |
| He et al. (2012) | 1 | 0 | 0.5 | 0 | 1 | 1 | 0 | 1 | 4.5 |
| Lien et al. (2012) | 1 | 1^l^ | 0.5 | 1^m^ | 1 | 1 | 1 | 1 | 7.5 |
| Darbandi et al. (2014) | 1 | 1^l^ | 0.5 | 1^n^ | 1 | 1 | 1 | 1 | 7.5 |
| Kim et al. (2014) | 1 | 1^o^ | 0 | 0 | 1 | 1 | 0 | 1 | 5 |
| Schukro et al. (2014) | 1 | 1^l^ | 1 | 0 | 1 | 1 | 1 | 1 | 7 |
| Yeo et al. (2014) | 1 | 1^l^ | 0.5 | 1^i^ | 1 | 1 | 0 | 1 | 6.5 |
| Yeh et al. (2015) | 1 | 1^l^ | 0.5 | 1^i^ | 1 | 1 | 1 | 1 | 7.5 |
| Hsu et al. (2016) | 1 | 0 | 0 | 0 | 0 | 1 | 0 | 1 | 3 |

Hsieh^1^: C.T. Hsieh; Hsieh^2^: C.H. Hsieh

^a^ Mentioned “ random” = 1 point; not mentioned = 0 point; ^b^ Described in the method, appropriate = 1 point; without description = 0 point; inappropriate = -1 point; ^c^ Double blind =1 point; single blind = 0.5 point; not blind = 0 point; ^d^ Described in the method, appropriate = 1 point; without description = 0 point; inappropriate = -1 point; ^e^ Reported the number or reason of dropout = 1 point; not reported = 0 point; ^f^ Clearly mentioned inclusion or exclusion criteria = 1 point; not reported = 0 point; ^g^ Adverse effect was recorded or reported = 1 point; not mentioned = 0 point; ^h^ Described in the method = 1 point; not reported = 0 point; ^i^ Using sealed envelopes; ^j^ By randomization number table; ^k^ Auricular acupuncture was performed and main outcome measures were determined by different persons; ^l^ By a computer-generated randomization number; ^m^ All stimulation devices were wrapped in skin-colored ventilation tape; ^n^ Intervention and placebo groups received the intervention in the similar way(ear plaster with or without seeds); ^o^ Participant pick up one number from a black box that contained small sheets numbered 1-58


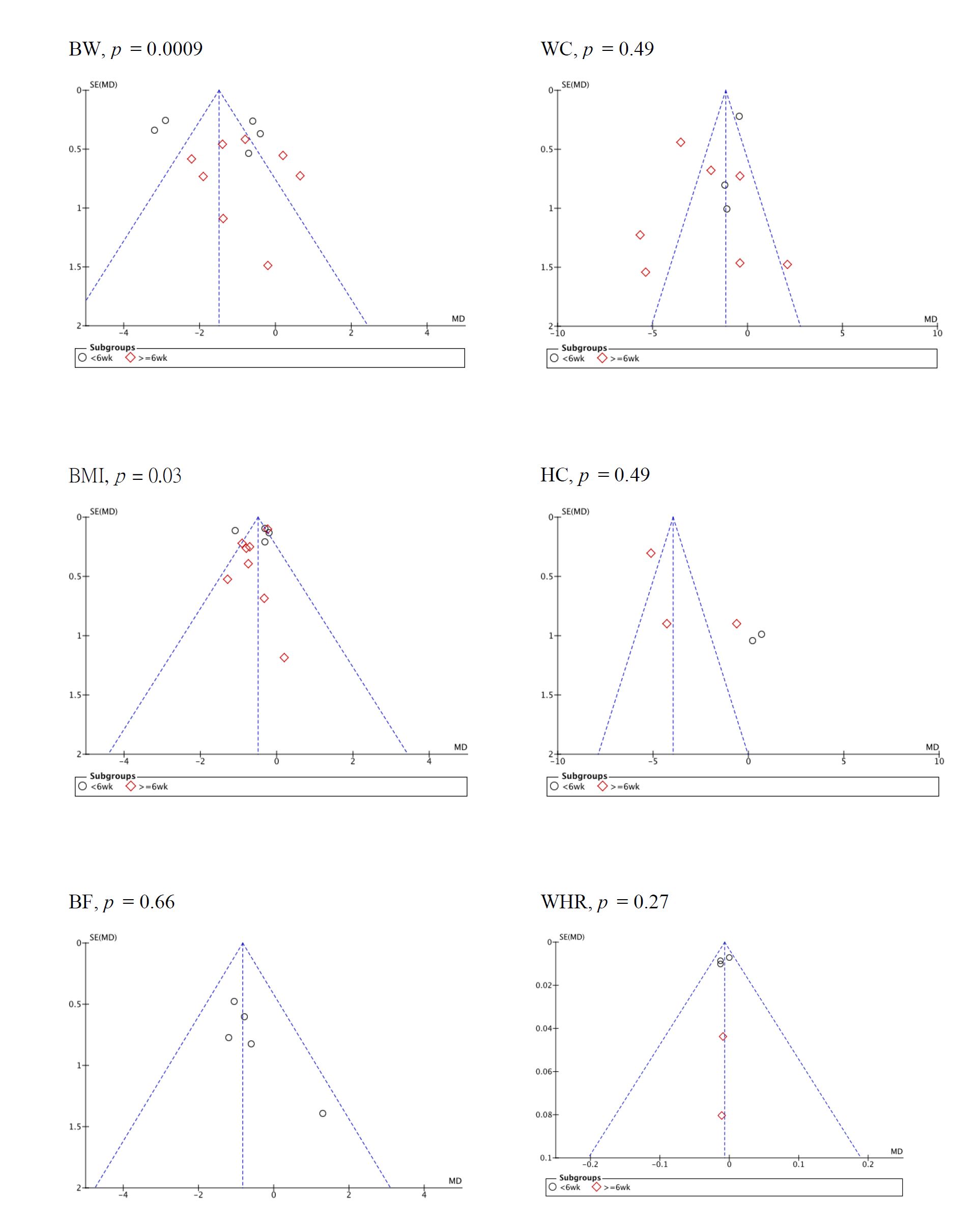


and Egger’s test to identify publication bias.

BW, body weight; BMI, body mass index; BF, body fat; WC, waist circumference; HC, hip circumference; WHR, waist-to-hip ratio.
